# Diagnostic Performance of a Molecular Test versus Clinician Assessment of Vaginitis

**DOI:** 10.1128/JCM.00252-18

**Published:** 2018-05-25

**Authors:** Jane R. Schwebke, Charlotte A. Gaydos, Paul Nyirjesy, Sonia Paradis, Salma Kodsi, Charles K. Cooper

**Affiliations:** a University of Alabama at Birmingham, Birmingham, Alabama, USA; b Johns Hopkins University, Baltimore, Maryland, USA; c Drexel University College of Medicine, Philadelphia, Pennsylvania, USA; d Becton, Dickinson and Company, BD Life Sciences—Diagnostic Systems, Quebec, QC, Canada; e Becton, Dickinson and Company, BD Life Sciences—Diagnostic Systems, Sparks, Maryland, USA; Marquette University

**Keywords:** Amsel's test, bacterial vaginosis, candidiasis, clinician diagnosis, molecular test, Nugent score, trichomoniasis, vaginitis, wet mount microscopy, diagnostic accuracy, sensitivity, specificity

## Abstract

Vaginitis is a common complaint, diagnosed either empirically or using Amsel's criteria and wet mount microscopy. This study sought to determine characteristics of an investigational test (a molecular test for vaginitis), compared to reference, for detection of bacterial vaginosis, Candida spp., and Trichomonas vaginalis. Vaginal specimens from a cross-sectional study were obtained from 1,740 women (≥18 years old), with vaginitis symptoms, during routine clinic visits (across 10 sites in the United States). Specimens were analyzed using a commercial PCR/fluorogenic probe-based investigational test that detects bacterial vaginosis, Candida spp., and Trichomonas vaginalis. Clinician diagnosis and in-clinic testing (Amsel's test, potassium hydroxide preparation, and wet mount) were also employed to detect the three vaginitis causes. All testing methods were compared to the respective reference methods (Nugent Gram stain for bacterial vaginosis, detection of the Candida gene *its2*, and Trichomonas vaginalis culture). The investigational test, clinician diagnosis, and in-clinic testing were compared to reference methods for bacterial vaginosis, Candida spp., and Trichomonas vaginalis. The investigational test resulted in significantly higher sensitivity and negative predictive value than clinician diagnosis or in-clinic testing. In addition, the investigational test showed a statistically higher overall percent agreement with each of the three reference methods than did clinician diagnosis or in-clinic testing. The investigational test showed significantly higher sensitivity for detecting vaginitis, involving more than one cause, than did clinician diagnosis. Taken together, these results suggest that a molecular investigational test can facilitate accurate detection of vaginitis.

## INTRODUCTION

Vaginitis is a frequent reason that women seek medical care; its accurate diagnosis is critical for appropriate treatment and for preventing recurrence (1). The three most common causes are bacterial vaginosis, vulvovaginal candidiasis, and trichomoniasis (2). Bacterial vaginosis is diagnosed based on Amsel's or Nugent criteria (3, 4). Criteria for vulvovaginal candidiasis include budding yeast or pseudohyphae on wet mount or positive culture with or without compatible clinical findings (5). Trichomoniasis is diagnosed through observation on wet mount or in culture or via biochemical detection through, antigen-, nucleic acid hybridization-, or nucleic acid amplification-based assays (5, 6).

Current standard of care testing relies heavily upon microscope equipment and training and requires certification per the Clinical Laboratory Improvement Act (7). In addition, the majority of real-world diagnoses are empirical and less than half of all treatments are based on objective assays (8), which can result in incorrect diagnosis and treatment (9). Molecular assays that target bacterial vaginosis, Candida spp., and Trichomonas vaginalis have the potential to improve diagnostic accuracy and reduce time to result compared to those for culture (10). This may be especially important for bacterial vaginosis, which involves multiple organisms of the vaginal microbiota (11).

The Food and Drug Administration-approved BD MAX vaginal panel (investigational test; Becton, Dickinson and Company, BD Life Sciences—Diagnostic Systems), using the BD MAX system, involves amplification-based DNA detection for all three common causes of vaginitis. This article provides results of additional analysis from a research study that was previously described by Gaydos et al. (12) in which sensitivity and specificity of at least 90% and 85%, respectively, were reported for bacterial, fungal, and protozoan causes. While the work by Gaydos et al. compared the investigational test to the reference methods for diagnostic performance in detection of vaginitis causes and included performance for both clinician-collected and self-collected samples, this study compared the clinician collected investigational test, in-clinic testing, and clinician diagnosis to reference methods defined as Nugent score for bacterial vaginosis and culture for both Candida spp. (followed by bidirectional sequencing) and Trichomonas vaginalis.

## MATERIALS AND METHODS

### Study design.

**FIG 1:**
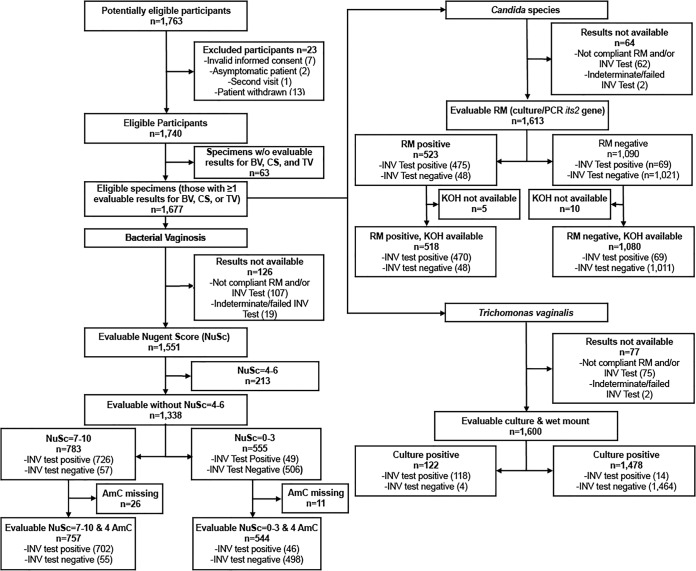
Evaluable specimens included in this study. Top left, eligible participants; bottom left, evaluable specimens for bacterial vaginosis; top right, evaluable specimens for Candida species; bottom right, evaluable specimens for Trichomonas vaginalis. Abbreviations: w/o, without; BV, bacterial vaginosis; CS, Candida species; TV, Trichomonas vaginalis; RM, reference method; INV, investigational, NuSc, Nugent score; AmC, Amsel's criteria; KOH, potassium hydroxide preparation.

The STARD statement was used to ensure accurate reporting in this article (13). The study design was a diagnostic accuracy, cross-sectional study that has been previously described (12). All eligible subjects were recruited consecutively between May and September 2015 if they reported symptoms of vaginitis (at least one of the following symptoms: abnormal vaginal discharge; painful or frequent urination; vaginal itching, burning, or irritation; painful or uncomfortable intercourse; and vaginal odor) during routine clinic visits. Institutional review board approval was obtained by all 10 participating centers, which were either academic medical center clinics or community clinics. Only specimens meeting study inclusion criteria were included in analyses (Fig. 1).

### Data collection.

The following vaginal swabs were obtained by a predetermined, rotating order of collection: one investigational test swab (BD MAX specimen collection swab; Becton, Dickinson and Company, BD Life Sciences—Diagnostic Systems; Sparks, MD), one cotton swab each for wet mount and Trichomonas vaginalis culture (Puritan Medical Products, Guilford, ME), and one BD liquid Amies elution swab collection and transport system (Becton, Dickinson and Company, BD Life Sciences—Diagnostic Systems) for Nugent scoring and Candida culture.

Data collection for this study was planned prior to performance of the index and reference tests. Results indicating test positivity for the reference methods were prespecified and were based on the presence or absence of vaginitis causes determined by the three assays described below. As previously used in the parent study (12), the reference method used in this study for bacterial vaginosis was Nugent scoring (4), the accepted gold standard, with score values of 0 to 3 (normal flora) or 7 to 10 (bacterial vaginosis) for bacterial vaginosis. For these analyses, only positive or negative scoring was considered; intermediate scores were not considered because no correlate result for intermediate is reported by the investigational test. Therefore, Amsel's criteria, used to resolve intermediate Nugent scores (4 to 6) (12), were not analyzed. For vulvovaginal candidiasis (all Candida spp. were combined), cultures were established, the current clinical standard for diagnosis, followed by bidirectional sequencing of the *its2* gene (from purified isolates). The InPouch TV culture system (Biomed Diagnostics, Inc.; White City, OR), incubated for 5 to 7 days (the latest recommended incubation time), was used as the reference method for trichomoniasis (14, 15).

### Investigational test.

The investigational test swab was stored in buffer during specimen transport to the laboratory. The investigational test (BD MAX vaginal panel; Becton, Dickinson and Company, BD Life Sciences—Diagnostic Systems) is a molecular test and was performed with the BD MAX system (Becton, Dickinson and Company, BD Life Sciences—Diagnostic Systems). The assay uses real-time PCR for amplification of specific DNA targets, followed by fluorogenic, target-specific probes to differentially detect bacterial vaginosis markers, including Lactobacillus spp. (Lactobacillus crispatus and L. jensenii), Gardnerella vaginalis, Atopobium vaginae, bacterial vaginosis-associated bacterium 2 (BVAB-2), and Megasphaera 1; Candida group (Candida albicans, C. tropicalis, C. parapsilosis, and C. dubliniensis), C. glabrata, and C. krusei; and Trichomonas vaginalis. For the purposes of this analysis, Candida group, C. glabrata, and C. krusei were combined (Candida spp.). Specimen turnaround time was approximately 3 h (including upfront processing time of less than 20 min) from initiation of testing to result. For bacterial vaginosis, the proprietary algorithm determined a positive or negative status based on the presence and concentration of each of the markers mentioned. For Candida spp. and Trichomonas vaginalis, test positivity was prespecified and determined by the presence or absence of target DNA (results reported as positive or negative).

### In-clinic tests.

As described previously (12), in-clinic tests were performed for bacterial vaginosis, Candida spp., and Trichomonas vaginalis. For bacterial vaginosis, Amsel's criteria were used (vaginal pH > 4.5; clue cells seen on wet mount microscopy; “whiff test”; and thin, homogeneous, grayish, or off-white vaginal discharge) (3). On wet mount microscopy, which was read by physicians or nurse practitioners (depending on the site), visualization of pseudohyphae or budding yeast was considered positive for vulvovaginal candidiasis. Visualization of motile trichomonads on wet mount microscopy was used to identify Trichomonas vaginalis. Further details of these methods have been described previously (12).

### Clinician diagnosis.

Overall, clinician diagnosis was based on clinical assessment of subject history, signs, and symptoms and was recorded in case report forms; findings from in-clinic testing (all in-clinic tests were utilized for the diagnosis) were also utilized.

Clinician investigators performed all the in-clinic tests and provided their diagnosis before obtaining any results from the reference methods or the investigational test. Results of the investigational test and reference methods were unknown, respective to each other, prior to their completion. The full study protocol may be accessed by contacting the corresponding author.

### Statistics.

Sensitivity, specificity, overall percent agreement (OPA), positive predictive value (PPV), and negative predictive value (NPV) were calculated according to standard equations. The confidence intervals (CI) were calculated using the Wilson score method (16). For the investigational test and in-clinic testing, within the 1,677 eligible specimens, results of not compliant for bacterial vaginosis (6.1%), Candida spp. (3.6%), and Trichomonas vaginalis (4.3%), or indeterminate/failed for bacterial vaginosis (1.1%), Candida spp. (0.1%), and Trichomonas vaginalis (0.1%), were not utilized for data analysis (Fig. 1).

Logistic modeling was performed to determine whether sensitivity and specificity were statistically different between the investigational test and either in-clinic testing or clinician diagnosis, relative to the reference method. A significance level of 0.05 was used. The statistical difference for OPA values was determined using the Cohen's kappa coefficient. The Wald confidence intervals are provided for the kappa statistic (17). A kappa statistic of > 0.90 indicates almost perfect agreement, 0.80 to 0.90 indicates strong agreement, 0.60 to 0.79 indicates moderate agreement, 0.40 to 0.59 indicates weak agreement, 0.21 to 0.39 indicates minimal agreement, and 0 to 0.20 indicates no agreement between the two populations being studied (beyond chance) (18). Test accuracy was determined by the formula (prevalence of vaginitis cause × test sensitivity) + (1 − prevalence of vaginitis cause × test specificity) (19). Sample size for this study was based on determinations described by Gaydos et al. (12).

## RESULTS

A total of 1,763 subjects were enrolled in the study, with 1,740 subjects completing study procedures (Fig. 1). Reasons for exclusion of 23 subjects included subject withdrawal (13), incorrect informed-consent process (7), enrollment of asymptomatic subject (2), and previous enrollment of subject in this study (1). Demographic data were described previously (12). Of the 1,740 subjects completing study procedures, 1,677 had evaluable specimens for at least one cause of vaginitis. For bacterial vaginosis, 1,338 subjects had a Nugent score of 0 to 3 or 7 to 10 (reference method). All four of Amsel's criteria were present for 1,301 subjects. For Candida spp., 1,613 subjects had an evaluable result with the reference method. Potassium hydroxide preparation results were also available for 1,598 of these subjects. For Trichomonas vaginalis, 1,600 subjects had culture (reference method) and wet mount results that were compliant and reportable. The age range for subjects in this study was 18 to 81 years (see Table S1 in the supplemental material).

**FIG 2:**
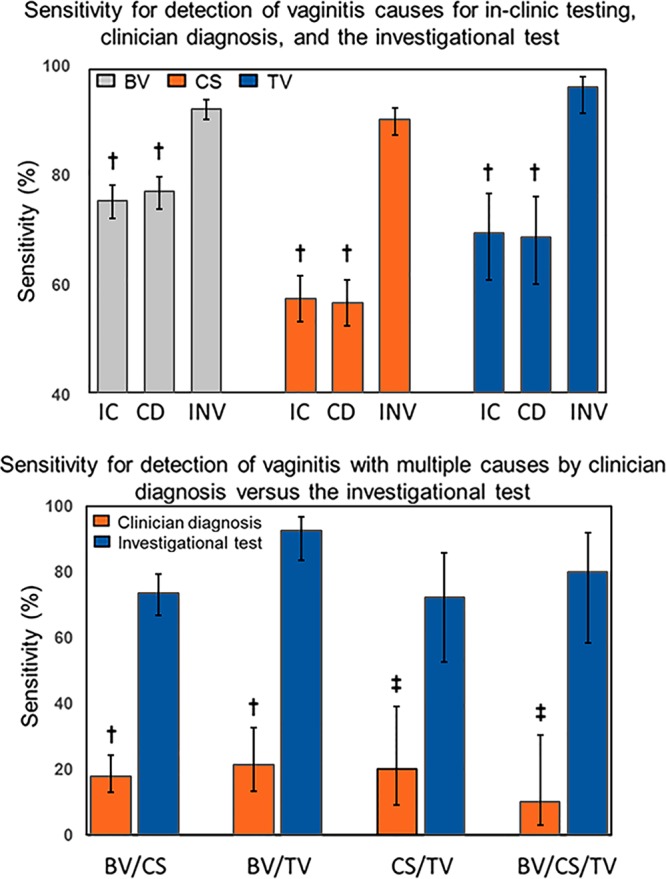
Sensitivity of diagnostic methods for detection of one or multiple causes of vaginitis. (Top) The sensitivity values (percent) for in-clinic testing, clinician diagnosis, and the investigational test are shown for bacterial vaginosis, Candida spp., and Trichomonas vaginalis. (Bottom) The sensitivity values (percent) for clinician diagnosis and the investigational test are shown for vaginitis cases involving more than one cause. Abbreviations: BV, bacterial vaginosis; CS, Candida spp.; TV, Trichomonas vaginalis; IC, in-clinic testing; CD, clinician diagnosis; INV, PCR-based molecular, investigational test. †, *P* < 0.0001; ‡, *P* < 0.0005.

Performance of the investigational test, Amsel's criteria (modified or original), and clinician diagnosis for detection of bacterial vaginosis was assessed by comparing their results to Nugent scores 0 to 3 and 7 to 10 (Table 1 and Fig. 2). Compared to the original Amsel's test, the investigational test resulted in a significantly higher sensitivity (75.6% versus 92.7%, respectively; *P* < 0.0001), with specificities of 94.1% and 91.5%, respectively. The modified Amsel's test (2/3), which omits discharge as a diagnostic criterion, had the highest sensitivity of all in-clinic tests but had a lower sensitivity (82.0%; *P* < 0.0001) than the investigational test; the modified Amsel's test (2/3) had a specificity (90.6%) that was similar to that of the investigational test. The investigational test had an OPA of 92.2% and a kappa value of 0.84. Whereas the original Amsel's test had a lower OPA of 83.3% (*P* < 0.0001) and a kappa value of 0.67, the modified Amsel's test (2/3; no discharge) also had a lower OPA (85.6%; *P* < 0.0001) compared to the investigational test and a kappa value of 0.71. The sensitivity of clinician diagnosis for bacterial vaginosis (77.3%) was significantly lower than that of the investigational test (*P* < 0.0001), whereas the specificities were similar (92.3% for clinician diagnosis). Clinician diagnosis had a lower OPA (83.6%; *P* < 0.0001) and kappa value (0.67) than did the investigational test (Table 1).

**TABLE 1:** Bacterial vaginosis: in-clinic test (individual or combination Amsel's criteria), investigational test, and clinician diagnosis versus Nugent score^*a*^

Parameter or test	% sensitivity (95% CI), no./total	% specificity (95% CI), no./total	% OPA (95% CI), no./total	Kappa value (95% CI)	% PPV (95% CI), no./total	% NPV (95% CI), no./total
pH	90.0 (87.6–91.9), 681/757	72.8 (68.9–76.4), 396/544	82.8 (80.6–84.7), 1,077/1,301	0.64 (0.60–0.68)	82.1 (79.4–84.6), 681/829	83.9 (80.3–86.9), 396/472
Discharge	58.9 (55.4–62.4), 446/757	90.1 (87.3–92.3), 490/544	71.9 (69.4–74.3), 936/1301	0.46 (0.42–0.50)	89.2 (86.2–91.6), 446/500	61.2 (57.8–64.5), 490/801
Clue cells	78.6 (75.5–81.4), 595/757	86.4 (83.3–89.0), 470/544	81.9 (79.7–83.9), 1,065/1,301	0.64 (0.59–0.68)	88.9 (86.3–91.1), 595/669	74.4 (70.8–77.6), 470/632
Whiff	77.1 (74.0–80.0), 584/757	94.3 (92.0–96.0), 513/544	84.3 (82.2–86.2), 1,097/1,301	0.69 (0.65–0.73)	95.0 (92.9–96.4), 584/615	74.8 (71.4–77.9), 513/686
Mod. Amsel (whiff/pH) (=2/2)^*b*^	74.2 (71.0–77.2), 562/757	95.8 (93.7–97.2), 521/544	83.2 (81.1–85.2), 1,083/1,301	0.67 (0.63–0.71)	96.1 (94.2–97.4), 562/585	72.8 (69.4–75.9), 521/716
Mod. Amsel (whiff/clue cell) (=2/2)^*b*^	72.1 (68.8–75.2), 546/757	95.6 (93.5–97.0), 520/544	81.9 (79.8–83.9), 1,066/1,301	0.65 (0.61–0.69)	95.8 (93.8–97.2), 546/570	71.1 (67.7–74.3), 520/731
Mod. Amsel (pH/clue cell) (=2/2)^*b*^	74.9 (71.7–77.9), 567/757	91.9 (89.3–93.9), 500/544	82.0 (79.8–84.0), 1,067/1,301	0.64 (0.60–0.68)	92.8 (90.5–94.6), 567/611	72.5 (69.0–75.7), 500/690
Mod. Amsel (no clue cell) (=2/3)^*c*^	79.8 (76.8–82.5), 604/757	91.2 (88.5–93.3), 496/544	84.6 (82.5–86.4), 1,100/1,301	0.69 (0.65–0.73)	92.6 (90.4–94.4), 604/652	76.4 (73.0–79.5), 496/649
Mod. Amsel (no whiff) (=2/3)^*c*^	80.3 (77.3–83.0), 608/757	88.4 (85.5–90.8), 481/544	83.7 (81.6–85.6), 1,089/1,301	0.67 (0.63–0.71)	90.6 (88.2–92.6), 608/671	76.3 (72.9–79.5), 481/630
Mod. Amsel (no pH) (=2/3)^*c*^	76.8 (73.6–79.6), 581/757	93.0 (90.6–94.9), 506/544	83.6 (81.4–85.5), 1,087/1,301	0.67 (0.64–0.71)	93.9 (91.7–95.5), 581/619	74.2 (70.8–77.3), 506/682
Mod. Amsel (no discharge) (=2/3)^*d*^	82.0 (79.1–84.6), 621/757^*e*^	90.6 (87.9–92.8), 493/544^*f*^	85.6 (83.6–87.4), 1,114/1,301^*e*^	0.71 (0.67–0.75)	92.4 (90.2–94.2), 621/672^*f*^	78.4 (75.0–81.4), 493/629^*e*^
Original Amsel (=3/4)	75.6 (72.4–78.5), 572/757^*e*^	94.1 (91.8–95.8), 512/544^*f*^	83.3 (81.2–85.2), 1,084/1,301^*e*^	0.67 (0.63–0.71)	94.7 (92.6–96.2), 572/604^*f*^	73.5 (70.1–76.6), 512/697^*e*^
Clinician diagnosis	77.3 (74.1–80.2), 585/757^*e*^	92.3 (89.7–94.4), 502/544^*f*^	83.6 (81.4–85.5), 1,087/1,301^*e*^	0.67 (0.63–0.71)	93.3 (91.2–94.9), 585/627^*f*^	74.5 (71.9–76.9), 502/674^*e*^
Investigational test	92.7 (90.7–94.4), 702/757	91.5 (88.9–93.6), 498/544	92.2 (90.7–93.6), 1,200/1,301	0.84 (0.81–0.87)	93.9 (91.9–95.4), 702/748	90.1 (87.3–92.3), 498/553

a Of the 1,677 eligible specimens, totals of 213 specimens with intermediate Nugent scores and 37 specimens with at least one Amsel's criterion missing were excluded to calculate the performance of the Amsel's criteria against Nugent scoring (0 to 3 and 7 to 10). Mod., modified.

b Only the tests indicated in parentheses were considered, and both needed to be positive.

c Four possible tests are considered: pH, discharge, clue cells, and whiff; tests in parentheses were not considered. Two out of the three remaining tests needed to be positive.

d Only pH, clue cells, and whiff were considered; discharge was not considered. Two of the three tests needed to be positive.

e *P* < 0.0001 compared to the investigational test.

f *P* > 0.05 compared to the investigational test.

Consistent with the relatively high sensitivity for the investigational test, the NPV for the investigational test was 90.1%, which was higher than those of the original Amsel test (73.5%; *P* < 0.0001), the modified Amsel test (2/3; no discharge) (78.4%; *P* < 0.0001), and clinician diagnosis (74.5%; *P* < 0.0001), respectively (Table 1). The PPV of the investigational test was higher than those of the other two methods, but no statistically significant difference was found. The prevalence of bacterial vaginosis in this study was 58%. Figure S1A contains likelihood ratios for comparison of PPV and NPV for the investigational test versus clinician diagnosis and in-clinic testing for bacterial vaginosis.

**FIG 3:**
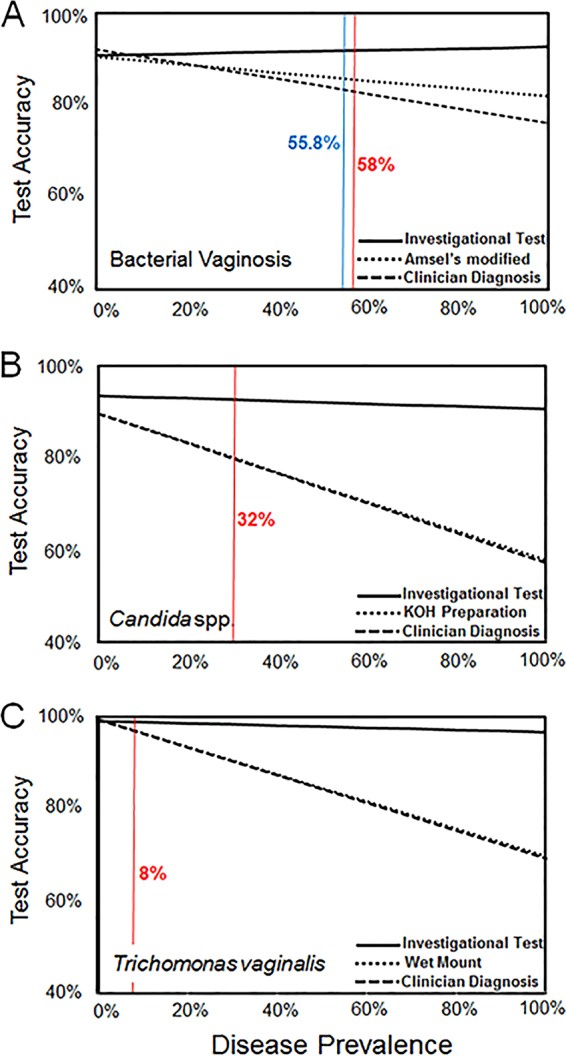
Test accuracy as a function of increasing prevalence of vaginitis cause. The three panels represent bacterial vaginosis (A), Candida spp. (B), and Trichomonas vaginalis (C). Change in test accuracy is plotted (*y* axis; 0% to 100%) as population prevalence changes (*x* axis; 0% to 100%). The actual prevalence in this study for each of the three causes in panels A to C is indicated with a vertical red line. The vertical blue line in (A) indicates the prevalence for bacterial vaginosis found in the study of Gaydos et al. (Nugent scoring 0 to 3 and 7 to 10 plus modified Amsel's criteria 2/3 without discharge for Nugent scoring 4 to 6; compared to Nugent in this study using 0 to 3 and 7 to 10) (12).

Figure 3A shows the change in accuracy of the investigational test, clinician diagnosis, and in-clinic testing as disease prevalence increases (from 0% to 100%). Clinician diagnosis and in-clinic testing show a decrease in accuracy with increasing prevalence, whereas the accuracy for the investigational test remains relatively constant. At very low disease prevalence, clinician diagnosis has a relatively high accuracy, which falls as prevalence values exceed 5%.

Performance of potassium hydroxide preparation (in-clinic test), clinician diagnosis, and the investigational test for detection of Candida spp. was assessed by comparing their results to those of the reference method (i.e., culture) (Table 2 and Fig. 2). The investigational test had a higher sensitivity (90.7% versus 57.5%, respectively; *P* < 0.0001) and a higher specificity (93.6% versus 89.4%, respectively; *P* < 0.0005) than did potassium hydroxide preparation. The sensitivity (56.8%; *P* < 0.0001) and specificity (89.2%; *P* = 0.0002) of clinician diagnosis for Candida spp. was significantly lower than that of the investigational test. The investigational test had a higher OPA (92.7%) than both potassium hydroxide preparation (79.0%; *P* < 0.0001) and clinician diagnosis (78.7%; *P* < 0.0001); the investigational test also had a higher corresponding kappa value (0.84) than did both potassium hydroxide preparation (0.50) and clinician diagnosis (0.49).

**TABLE 2:** Candida in-clinic test, investigational test, and clinician diagnosis versus Candida culture^*a*^

Test	% sensitivity (95% CI), no./total	% specificity (95% CI), no./total	OPA (95% CI), no./total	Kappa value (95% CI)	% PPV (95% CI), no./total	% NPV (95% CI), no./total
KOH preparation overall	57.5 (53.2–61.7), 298/518^*b*^	89.4 (87.4–91.1), 965/1,080^*c*^	79.0 (77.0–81.0), 1,263/1,598^*b*^	0.50 (0.45–0.54)	72.2 (67.6–76.3), 298/413^*b*^	81.4 (79.1–83.5), 965/1,185^*b*^
Clinician diagnosis	56.8 (52.5–61.0), 294/518^*b*^	89.2 (87.2–90.9), 963/1,080^*c*^	78.7 (76.6–80.6), 1,257/1,598^*b*^	0.49 (0.44–0.53)	71.5 (67.0–75.7), 294/411^*b*^	81.1 (78.8–83.3), 963/1,187^*b*^
Investigational test	90.7 (87.9–92.9), 470/518	93.6 (92.0–94.9), 1,011/1,080	92.7 (91.3–93.9), 1,481/1,598	0.84 (0.81–0.86)	87.2 (84.1–89.8), 470/539	95.5 (94.0–96.6), 1,011/1,059

a Fifteen specimens were excluded because KOH preparation results were unavailable. KOH, potassium hydroxide.

b *P* < 0.0001 compared to the investigational test.

c *P* < 0.0005 compared to the investigational test.

As shown in Table 2 and in Fig. S1B, the PPV and NPV of the investigational test (87.2% and 95.5%, respectively) were significantly greater than those for potassium hydroxide preparation (72.2% [*P* < 0.0001] and 81.4% [*P* < 0.0001], respectively) and clinician diagnosis (71.5% [*P* < 0.0001] and 81.1% [*P* < 0.0001], respectively). Potassium hydroxide preparation and clinician diagnosis accuracy both decreased with increasing prevalence, whereas the accuracy of the investigational test remained high (Fig. 3B). The population prevalence of Candida spp. in the study was 32%.

Table 3 demonstrates the comparative performance for the detection of Trichomonas vaginalis with wet mount (in-clinic test), clinician diagnosis, and the investigational test compared to that of culture (reference method). The investigational test had a sensitivity of 96.7% for Trichomonas va*ginalis*, which was statistically greater than that for wet mount (69.7%; *P* < 0.0001) and clinician diagnosis (68.9%; *P* < 0.0001 [Fig. 2]), whereas no statistical difference was found for the specificity of the investigational test (99.1%) versus wet mount (99.5%; *P* = 0.1336) or clinician diagnosis (99.1%; *P* = 0.8273). The investigational test had a significantly greater OPA (98.9%, versus 97.2% and 96.8%, respectively; *P* < 0.0001 for both comparisons) and a higher kappa value (0.92 versus 0.78 and 0.75, respectively) than wet mount and clinician diagnosis.

**TABLE 3:** Trichomonas vaginalis: in-clinic test, investigational test, and clinician diagnosis versus culture

Test	% sensitivity (95% CI), no./total	% specificity (95% CI), no./total	% OPA (95% CI), no./total	Kappa value (95% CI)	% PPV (95% CI), no./total	% NPV (95% CI), no./total
Wet mount	69.7 (61.0–77.1), 85/122^*a*^	99.5 (98.9–99.7), 1,470/1,478^*b*^	97.2 (96.3–97.9), 1,555/1,600^*a*^	0.78 (0.71–0.84)	91.4 (83.9–95.6), 85/93^*b*^	97.5 (96.6–98.2), 1,470/1,507^*a*^
Clinician diagnosis	68.9 (60.2–76.4), 84/122^*a*^	99.1 (98.5–99.5), 1,465/1,478^*b*^	96.8 (95.8–97.6), 1,549/1,600^*a*^	0.75 (0.69–0.82)	86.6 (78.4–92.0), 84/97^*b*^	97.5 (96.6–98.2), 1,465/1,503^*a*^
Investigational test	96.7 (91.9–98.7), 118/122	99.1 (98.4–99.4), 1,464/1,478	98.9 (98.2–99.3), 1,582/1,600	0.92 (0.89–0.96)	89.4 (83.0–93.6), 118/132	99.7 (99.3–99.9), 1,464/1,468

a *P* < 0.0001 compared to the investigational test.

b *P* > 0.05 compared to the investigational test.

As shown in Table 3 and in Fig. S1C, the PPV for the investigational test was 89.4%, compared to 86.6% for clinician diagnosis and 91.4% for wet mount. The NPV for the investigational test (99.7%) was greater than for wet mount (97.5%; *P* < 0.0001) and clinician diagnosis (97.5%; *P* < 0.0001). Wet mount and clinician diagnosis accuracy both decreased with increasing prevalence, whereas the investigational test accuracy remained relatively high and constant over increasing prevalence (Fig. 3C). The population prevalence of Trichomonas vaginalis in the study was 8%.

Table 4 shows the percentages in cases involving coinfection for vaginitis detected by the investigational test and clinician diagnosis. The investigational test had greater sensitivity than clinician diagnosis for bacterial vaginosis and Candida spp. (73.5% versus 17.8%; *P* < 0.0001), bacterial vaginosis and Trichomonas vaginalis (92.4% versus 21.2%; *P* < 0.0001), and all three causes combined (80% versus 10%; *P* < 0.0005). Twenty-three cases of coexisting Candida spp. and Trichomonas vaginalis were detected with the investigational test, whereas seven cases were detected with clinician diagnosis. Figure S1D to G show the likelihood ratios of the investigational test compared to clinician diagnosis and reflect the consistently high sensitivity of the investigational test compared to that of clinician diagnosis (Fig. 2).

**TABLE 4:** Performance of the investigational test and clinician diagnosis for detection of vaginitis with single or multiple causes^*f*^

Cause	Method	% of cases (no.) (*n* = 1,264)^*e*^	% sensitivity (95% CI), no./total	% specificity (95% CI), no./total	% PPV (95% CI), no./total	% NPV (95% CI), no./total
BV	CD	48.3 (611)	76.8 (73.6–79.7), 566/737^*a*^	91.5 (88.8–93.6), 482/527	92.6 (90.3–94.5), 566/611	73.8 (70.3–77.0), 482/653^*a*^
	INV test	57.8 (731)	92.8 (90.7–94.5), 684/737	91.1 (88.3–93.2), 480/527	93.6 (91.6–95.1), 684/731	90.1 (87.2–92.3), 480/533
Candida spp.	CD	25.5 (322)	56.9 (52.0–61.7), 227/399^*a*^	89.0 (86.8–90.9), 770/865^*a*^	70.5 (65.3–75.2), 227/322^*a*^	81.7 (79.1–84.1), 770/942^*a*^
	INV test	32.2 (407)	90.2 (86.9–92.8), 360/399	94.6 (92.8–95.9), 818/865	88.5 (85.0–91.2), 360/407	95.4 (93.8–96.7), 818/857
TV	CD	5.3 (67)	68.6 (58.2–77.4), 59/86^*a*^	99.3 (98.7–99.7), 1,170/1,178	88.1 (78.2–93.8), 59/67	97.7 (96.7–98.4), 1,170/1,197^*a*^
	INV Test	7.5 (95)	96.5 (90.2–98.8), 83/86	99.0 (98.2–99.4), 1,166/1,178	87.4 (79.2–92.6), 83/95	99.7 (99.2–99.9), 1,166/1,169
BV/Candida spp.	CD	4.7 (59)	17.8 (13.0–24.0), 33/185^*a*^	97.6 (96.5–98.4), 1,053/1,079^*c*^	55.9 (43.3–67.8), 33/59^*d*^	87.4 (85.4–89.1), 1,053/1,205^*a*^
	INV test	15.0 (189)	73.5 (66.7–79.3), 136/185	95.1 (93.6–96.2), 1,026/1,079	72.0 (65.2–77.9), 136/189	95.4 (94.0–96.5), 1,026/1,075
BV/TV	CD	1.4 (18)	21.2 (13.1–32.5), 14/66^*a*^	99.7 (99.1–99.9), 1,194/1,198^*d*^	77.8 (54.8–91.0), 14/18	95.8 (94.6–96.8), 1,194/1,246^*a*^
	INV test	6.0 (76)	92.4 (83.5–96.7), 61/66	98.7 (97.9–99.2), 1,183/1,198	80.3 (70.0–87.7), 61/76	99.6 (99.0–99.8), 1,183/1,188
Candida spp./TV	CD	0.6 (7)	20.0 (8.9–39.1), 5/525^*b*^	99.8 (99.4–100), 1,237/1,239	71.4 (35.9–91.8), 5/7	98.4 (97.6–99.0), 1,237/1,257^*b*^
	INV test	1.8 (23)	72.0 (52.4–85.7), 18/25	99.6 (99.1–99.8), 1,234/1,239	78.3 (58.1–90.3), 18/23	99.4 (98.8–99.7), 1,234/1,241
BV/Candida spp./TV	CD	0.2 (3)	10.0 (2.8–30.1), 2/20^*b*^	99.9 (99.5–100), 1,243/1,244	66.7 (20.8–93.9), 2/3	98.6 (97.8–99.1), 1,243/1,261^*b*^
	INV test	1.6 (20)	80.0 (58.4–91.9), 16/20	99.7 (99.2–99.9), 1,240/1,244	80.0 (58.4–91.9), 16/20	99.7 (99.2–99.9), 1,240/1,244

a *P* < 0.0001 compared to the investigational test.

b *P* < 0.0005 compared to the investigational test.

c *P* < 0.005 compared to the investigational test.

d *P* < 0.05 compared to the investigational test.

e Each specimen in this subset (*n* = 1,264) was included if and only if it had reportable results for all three reference methods (each reference method for BV, Candida spp., and TV), reportable results for the INV test, and reportable results for CD. Data for in-clinic testing were not included here, nor were they considered in the performance calculations.

f BV, bacterial vaginosis; CD, clinician diagnosis; INV, investigational; TV, Trichomonas vaginalis.

## DISCUSSION

The investigational molecular test used in this study is the first Food and Drug Administration-cleared nucleic acid amplification test for detection of the three major causes of vaginitis: bacterial vaginosis, Candida spp., and Trichomonas vaginitis. For these three causes, the investigational test consistently outperformed in-clinic testing and clinician diagnosis for sensitivity, with no depreciation in specificity (Tables 1 to 3 and Fig. 2 and 3). Importantly, the investigational test had the highest OPA with the reference test and better NPV for all causes compared to in-clinic testing and clinician diagnosis. Finally, the investigational test resulted in high diagnostic accuracy and likelihood ratios across all three vaginitis causes.

Traditionally, a diagnosis of vaginitis has been performed through clinical findings, medical history, and in-clinic testing, with the last representing an essential component for the establishment of a clinician diagnosis. For bacterial vaginosis, some combination of the Amsel's criteria is the mainstay for standard of care diagnosis in the clinic. CDC guidelines (5) suggest that three out of four Amsel's criteria should be positive (Amsel's original). However, Amsel's criteria are known to be highly subjective and open to interpretation (20, 21). In the current study, of all Amsel's components, pH had the highest sensitivity, while the whiff test had the highest specificity (Table 1). Other studies have reported that the presence of clue cells is the key pathognomonic feature of bacterial vaginosis, but this requires high technical expertise and appropriate laboratory infrastructure (22). Also, previous data showed better agreement between the Nugent score and Amsel's criteria when the latter did not include vaginal discharge as a criterion (12, 23). Our findings confirm this, as we showed that removing discharge as a criterion and looking for two out of three positive Amsel's criteria (modified Amsel 2/3 without discharge) improved test sensitivity, NPV, and OPA compared to Amsel's original testing. In this study, clinician diagnosis reported an OPA that matched better with the Amsel's original test than the modified Amsel 2/3 without discharge (Table 1). This suggests that clinician diagnosis in our study likely involved Amsel's original test. When considering applicability, it should be noted that Amsel's criteria were applied during this study within a highly controlled research environment involving consistent prestudy and ongoing training and quality monitoring; this may not accurately reflect the empirical nature of Amsel's criteria performance as typically used in clinical practice.

We determined the accuracy for detecting vaginitis from the three testing methods. The empirical accuracy for all three diagnostic methods depends on several factors, including test performance, prevalence, and the actual cause of vaginitis. For all three vaginitis causes, this report shows that as prevalence values increase, the accuracy of the investigational test remains relatively high and constant, while the accuracy for clinician diagnosis decreases (Fig. 3). However, this conclusion assumes that the operation characteristics of in-clinic testing and clinician diagnosis do not change at high prevalence values for vaginitis causes, which may not be the case.

Consistent with diagnosis of vaginitis by single causes, the investigational test outperformed clinician diagnosis of vaginitis that was due to multiple causes. The investigational test was more sensitive and had relatively high likelihood ratios for vaginitis with multiple causes (Table 4; see also Fig. S1D to G). It has previously been observed that the sensitivity of Amsel's criteria is diminished when Trichomonas vaginalis or Candida spp. are also present (24). They could explain the drastic drop in diagnostic performance that occurred for clinician diagnosis from single to multiple causes. Our results suggest that the investigational test may be resistant to reductions in diagnostic sensitivity when multiple causes of vaginitis are present.

This study had limitations that prevent an exact interpretation of the findings. Several analyses presented here involved observations for each type of infection that were excluded due to noncompliance or inability to report. It is possible, for example, that listing these types of observations as “not compliant” or “not reportable” for the investigational test, in lieu of “failure to correctly diagnose,” may have artificially improved its operating characteristics. Other limitations include the fact that the investigational assay may have resulted in an overdiagnosis of vaginitis, as it cannot distinguish nonpathogenic colonization from pathogenic growth; this would be considered for clinician diagnosis (25, 26). However, the clinical cutoff for the investigational test was set by the current reference standard for diagnosing Candida spp. (positive fungal culture report), and therefore, the results are consistent with everyday practice. Moreover, bacterial vaginosis may be detected by the Nugent score (7 to 10) but also be asymptomatic (27). The investigational test showed the best agreement with the Nugent score, which is the gold standard, but may have included asymptomatic bacterial vaginosis. The bacterial vaginosis algorithm for the investigational test was set by the composite reference method of concordant positive and negative Nugent and Amsel's criteria. Therefore, only unambiguous specimens for bacterial vaginosis status were used to develop the algorithm. Additionally, this study employed a cross-sectional design that did not evaluate clinical outcomes for patients with discordant reference method results and clinician diagnosis. Only clinics with expertise and resource availability for detection of the four Amsel's criteria and wet mount procedures were chosen as study sites. Therefore, clinician diagnosis benefited from reliability of in-clinic results in a way that might not occur under real-life conditions. Thus, the actual difference in clinician diagnosis versus the investigational test may likely be greater than that seen in this study. Finally, in this study we omitted the intermediate values for Nugent scoring (4 to 6, as described in Materials and Methods), whereas Gaydos et al. used the composite reference method of Nugent score combined with the modified Amsel 2/3 criteria without discharge to discriminate intermediate Nugent scoring (4 to 6). We may have missed some cases of bacterial vaginosis, the exclusion of which could have led to either an over- or underestimation of performance in the investigational test. However, the prevalence of bacterial vaginosis in this study (58%) was very close to that reported by Gaydos et al. (55.8%) (12).

The results from the current study support the potential utility of the investigational test in the differential diagnosis of vaginitis (28). While some laboratory tests take 2 to 7 days to provide results, the investigational test results are generally available within 24 h. Although future work is required to establish the cost/benefit ratio regarding the application of this investigational test in a practical setting, its high sensitivity, specificity, and accuracy (across a large spectrum of disease prevalence) should impart benefits and decrease the chance of needless treatment of patients that are negative for the disease (29). This may prove especially important with cases of vaginitis that involve multiple causes, where the sensitivity of clinician diagnosis may be limited.

## Supplementary Material

Supplemental material
