# Supplementary material for: Diagnostic Performance of a Molecular Test versus Clinician Assessment of Vaginitis
Source: J Clin Microbiol. 2018 May 25;56(6):e00252-18. doi: 10.1128/JCM.00252-18 (PMC5971525; doi:10.1128/JCM.00252-18)
Supplement: Supplemental material [file supp_56_6_e00252-18__index.html]

Supplemental material 

# Diagnostic Performance of a Molecular Test versus Clinician Assessment of Vaginitis

## Supplemental material

- Supplemental file 1 -

  Fig. S1 (Plotted likelihood ratios)

  PDF, 123K
- Supplemental file 2 -

  Table S1 (Demographic information and medical history of the study population)

  PDF, 34K
