# Supplementary material for: Diagnostic Performance of a Molecular Test versus Clinician Assessment of Vaginitis
Source: J Clin Microbiol. 2018 May 25;56(6):e00252-18. doi: 10.1128/JCM.00252-18 (PMC5971525; doi:10.1128/JCM.00252-18)
Supplement: Supplemental material [file JCM.00252-18_zjm999095976s1.pdf]

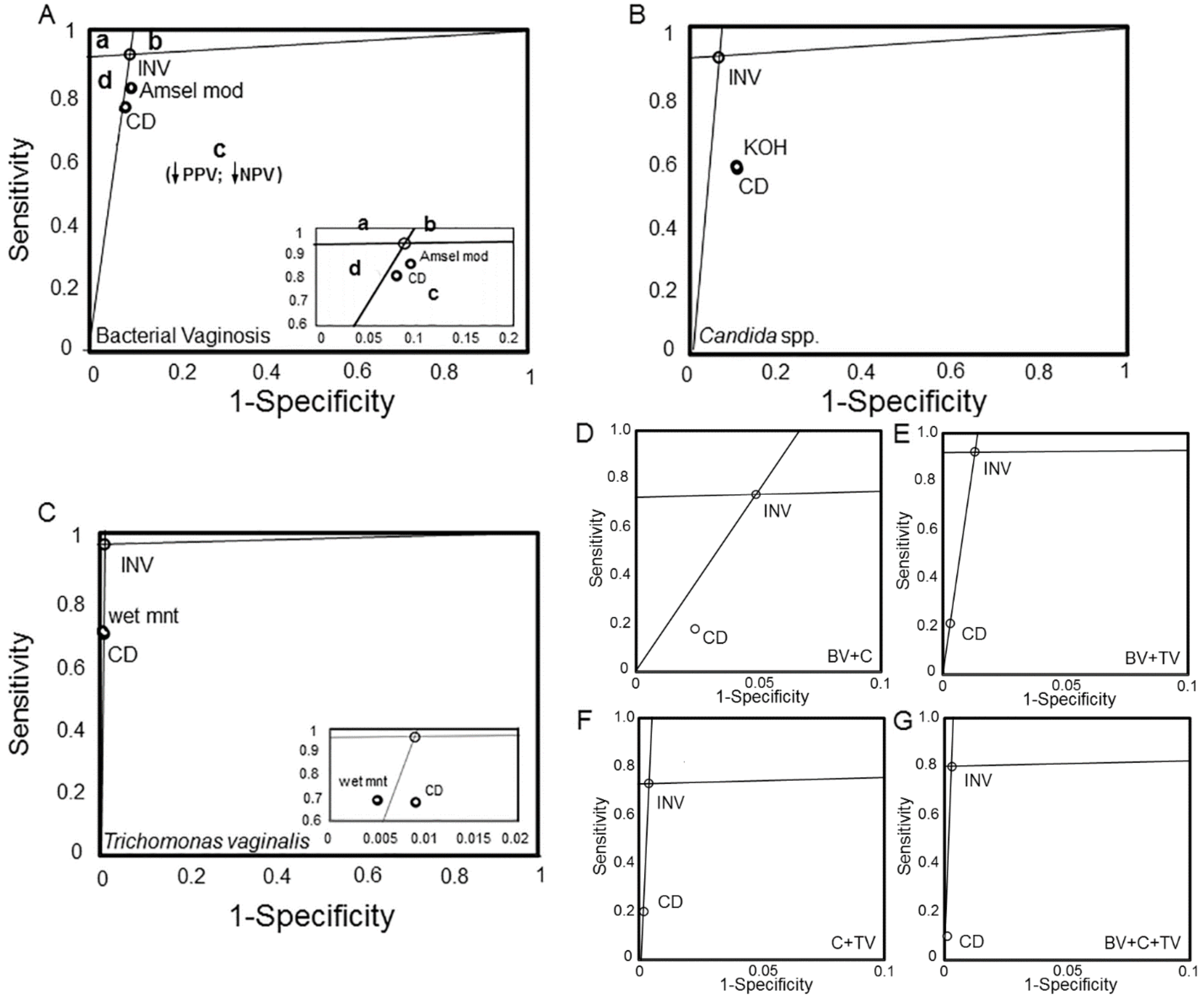

Figure S1. Plotted likelihood ratios (1-specificity against sensitivity) for the investigational test. The panels represent bacterial vaginosis (A; LR+ of 10.2, 8.7, and 9.4 for the investigational test, the Amsel's modified test, and clinician diagnosis, respectively), *Candida* spp. (B, LR+ of 14.2, 5.4, and 5.3 for the investigational test, the Amsel's modified test, and clinician diagnosis, respectively), and *Trichomonas vaginalis* (C, LR+ of 107.4, 139.4, and 76.6 for the investigational test, the Amsel's modified test, and clinician diagnosis, respectively). Relative PPV and NPV results of in-clinic tests and clinician diagnoses can be compared in four areas relative to the investigational test. Area (a) represents tests with a PPV and NPV value > investigational test; (b) represents PPV < and NPV > investigational test; (c) represents PPV and

NPV < investigational test; (d) represents PPV > and NPV< investigational test. Inset panels in (A) and (C) show enlarged views of the intersection over the likelihood ratio of the investigational test. Panels D-G, sensitivity to 1-specificity plots for the investigational test and clinician diagnosis for detection of cases of vaginitis with multiple causes; (D) bacterial vaginosis and *Candida* spp. (+LR: 7.4 and 15 for CD and INV, respectively), (E) bacterial vaginosis and *Trichomonas vaginalis* (+LR: 63.5 and 73.8 for CD and INV, respectively), (F) *Candida* spp. and *Trichomonas vaginalis* (+LR: 123.9 and 178.4 for CD and INV, respectively), and (G) bacterial vaginosis, *Candida* spp. and *Trichomonas vaginalis* (+LR: 124.4 and 248.8 for CD and INV, respectively). Abbreviations: LR+, positive likelihood ratio; PPV, positive predictive value; NPV, negative predictive value; INV, investigational test; CD, clinician diagnosis; Amsel mod Amsel's modified test (2/3 indicators); KOH, potassium hydroxide preparation; wet mnt, wet mount preparation; BV, bacterial vaginosis, C, *Candida* spp.; TV, *Trichomonas vaginalis*
