# Supplementary material for: Diagnostic Performance of a Molecular Test versus Clinician Assessment of Vaginitis
Source: J Clin Microbiol. 2018 May 25;56(6):e00252-18. doi: 10.1128/JCM.00252-18 (PMC5971525; doi:10.1128/JCM.00252-18)
Supplement: Supplemental material [file JCM.00252-18_zjm999095976s2.pdf]

Table S1. Demographic information and medical history of the study population (N=1,677).

**Characteristic**	
Mean age (SD)	29.3 (9.4)
Median age (Min, Max)	27 (18, 81)
Region (USA)	
East	37.4% (627)
Center, south	37.3% (626)
Center, north	9.8% (164)
West	15.5% (260)
Clinic type	
STD or HIV	18.3% (307)
Family planning	66.6% (1,117)
Ob/Gyn	15.1% (253)
Race/Ethnicity	
Asian	3.6% (61)
Black/African American	53.2% (892)
Caucasian	25.0% (419)
Other[†]	9.4% (158)
Hispanic/Latino	8.8% (147)
Age (years)	
18-29	63.2% (1,060)
30-39	22.4% (375)
40-49	10.2% (171)
≥50	4.2% (71)
Education	
<High school	3.4% (57)
High school (or equivalent)	30.1% (505)
>High school	64.0% (1,073)
Unknown	2.5% (42)
Sexual partners in the past year	
≤1	53.2% (893)
2-3	35.4% (594)
≥4	9.1% (153)
Declined to answer	2.2% (37)
HIV status	
Seronegative	83.5% (1,401)
Seropositive	1.0% (17)
Unknown	15.3% (257)
Not available	0.1% (2)
Type of symptom	
Abnormal vaginal discharge	74.5% (1,249)
Painful or frequent urination	11.7% (197)
Vaginal itching, burning, or irritation	51.6% (866)
Painful/uncomfortable intercourse	10.1% (169)
Vaginal odor	48.2% (809)
Number of symptoms	
1	32.2% (540)
2	44.9% (753)
3	18.1% (303)
4	4.2% (70)
5	0.7% (11)
Exposure to medications	
Oral antibiotics	11.7% (196)
Vaginal antibiotics	2.9% (48)
Antifungals	8.8% (148)

Abbreviations: SD, standard deviation; Min, minimum age; Max, maximum age; USA, United States of America; STD, sexually transmitted disease; HIV, human immunodeficiency virus; Ob/Gyn, obstetrics and gynecology

[†]Includes Native Hawaiian/other Pacific Island, American Indian or Alaskan native, mixed ethnicity, or declined to answer/unknown
